# Disseminated abscesses due to *Mycobacterium wolinskyi* and *Mycobacterium mageritense*: An unusual mixed infection

**DOI:** 10.1016/j.jdcr.2025.04.024

**Published:** 2025-06-06

**Authors:** Verena Gerlinde Frings, Thiên-Trí Lâm, Christoph Lange, Matthias Goebeler, Johanna Stoevesandt

**Affiliations:** a Department of Dermatology, Venereology and Allergology, University Hospital Würzburg, Würzburg, Germany; b Institute for Hygiene and Microbiology, University of Würzburg, Würzburg, Germany; c Division of Clinical Infectious Diseases, Research Center Borstel, Borstel, Germany; d German Center for Infection Research (DZIF), Germany; e International Health/Infectious Diseases, University of Lübeck, Lübeck, Germany; f Department of Medicine, Karolinska Institute, Stockholm, Sweden; g Department of Medicine, University of Namibia School of Medicine, Windhoek, Namibia

**Keywords:** 16S rRNA, immunosuppression, interferon-gamma, Mendelian susceptibility, rapidly growing mycobacteria

## Introduction

We present a case of disseminated abscesses caused by *Mycobacterium wolinskyi* and *Mycobacterium mageritense* in a seemingly immunocompetent individual with no identifiable predisposing factors. Nontuberculous mycobacterial infections represent an infrequent, though possibly underestimated differential diagnosis of cutaneous abscesses unresponsive to standard antibiotic treatment. Genetic defects affecting the interferon-gamma (IFNγ)/interleukin-12 axis and autoantibodies to IFNγ have been described as predisposing factors for active clinical infections with weakly virulent mycobacteria in apparently immunocompetent hosts.^1^^,^^2^ The genetically closely related species *M. wolinskyi* and *M. mageritense* have been recognized as an occasional cause of surgical site infections.^3^, ^4^, ^5^

## Case report

A 46-year-old woman of Asian origin presented to our department with a 2-week history of painful cutaneous nodules accompanied by fever up to 39 °C. Lesions had initially appeared on the chin, subsequently spread to the cheeks and back of the hands, and were unresponsive to intravenous antibiotic treatment with cefuroxime. She declared to be otherwise healthy, denied an increased susceptibility to infections in the past and was not on any medication. She denied any preceding surgical, dental, or cosmetic interventions, or acupuncture. About 1 week prior to the onset of symptoms, she had cleaned her husband’s aquarium without wearing gloves. No other family members were affected.

**Fig 1:**
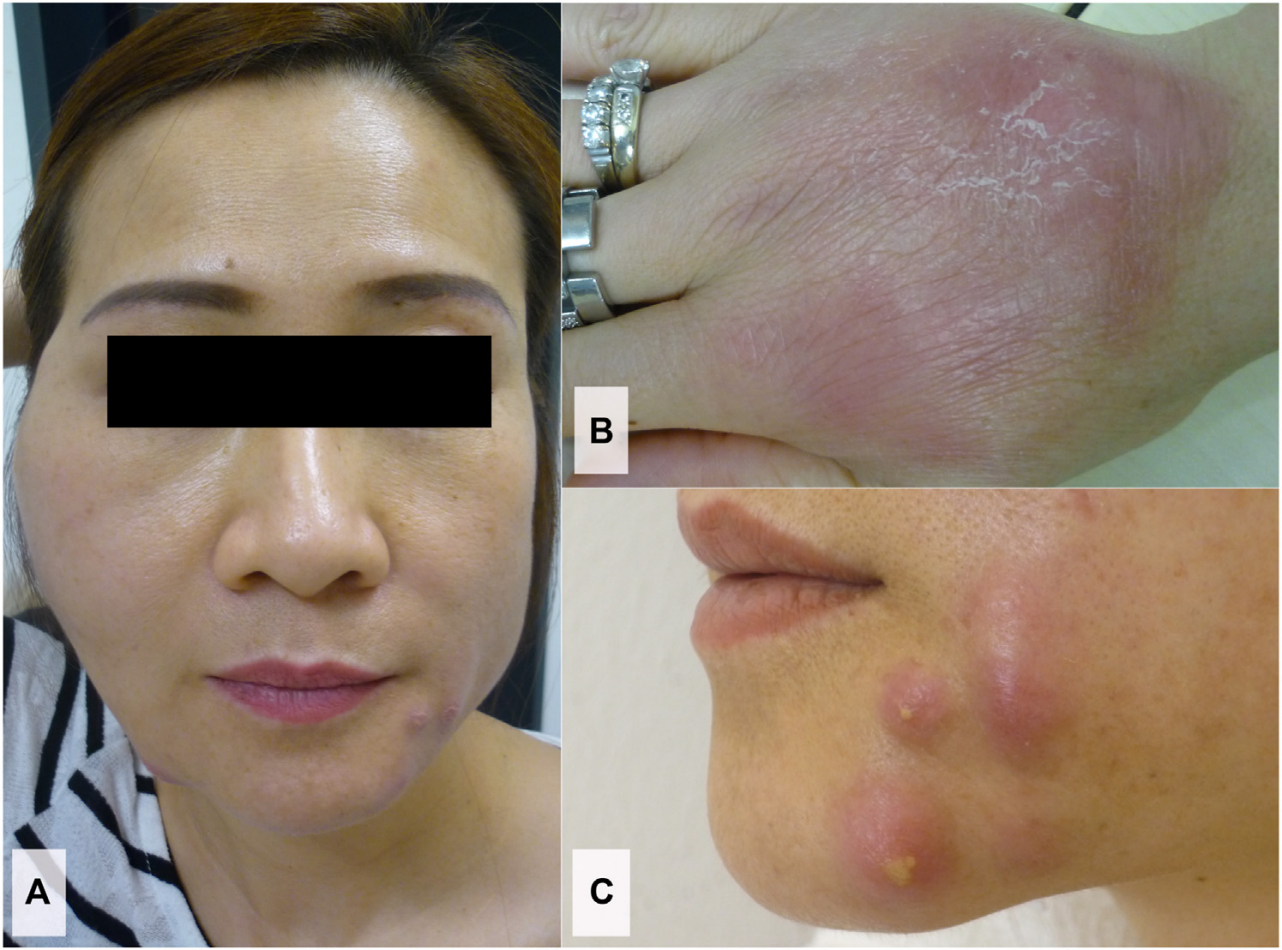
Cutaneous abscesses due to a mixed infection with *M. wolinskyi* and *M. mageritense*. Extensive subcutaneous swelling with palpable fluctuation of the right cheek (**A**). Erythematous nodules of 1-5 centimeters in diameter on the dorsum of the right hand (**B**), and over the mandibula (**C**).

Clinical examination on admission showed numerous erythematous cutaneous nodules of 1-5 centimeters in diameter (Fig 1) and a painless enlargement of the cervical lymph nodes. Mouth opening was limited due to extensive swelling of the right cheek. Laboratory tests revealed a pronounced leukocytosis (19,300 cells/μL), and a moderately elevated C-reactive protein (3.6 mg/dL; reference value < 0.5 mg/dl). Blood cultures were negative. Serological examination for human immunodeficiency virus was negative. Microscopic examination of pus revealed partially stained gram-positive rods, which proved acid-fast upon Ziehl-Neelsen staining. Nucleic acid amplification tests for *M. tuberculosis*-specific 16S rRNA were negative; and no *M. tuberculosis*-reactive T lymphocytes were detected in the Interferon Gamma Release Assay. Cultures from pus aspirates on Columbia Blood Agar and modified Middlebrook 7H9 Broth in a Mycobacteria Growth Indicator Tube system revealed bacterial growth within 5 days. 16S rRNA polymerase chain reaction and subsequent analysis of the 800 base pairs amplification product showed sequences of 2 different *Mycobacterium* spp. Alignment of both sequences confirmed a mixed infection with *M. wolinskyi* and *M. mageritense*. Growth of *M. fortuitum* was detected in samples of aquarium water but considered unrelated to the patient’s infection. Abnormalities in the IFN-γ/signal transducer and activator of transcription protein 1 signaling pathway could be excluded, as signal transducer and activator of transcription protein 1 phosphorylation was not impaired after stimulation of patient monocytes with lipopolysaccharide and IFN-γ. IFN-γ production after stimulation with Concanavalin A was within normal range.

All abscesses were incised, rinsed and drained. A quadruple therapy comprising intravenous amikacin (1 g/d), oral linezolid (600 mg/d), oral doxycycline (200 mg/d), and oral moxifloxacin (400 mg/d) led to complete clearance of cutaneous lesions within 12 weeks. Subsequent oral triple therapy with linezolid, doxycycline, and moxifloxacin was carried out for another 3 months. 8 months after completing treatment, she was confirmed to be in continued remission.

## Discussion

Both *M. wolinskyi* and *M. mageritense* are nontuberculous rapidly growing mycobacteria (RGM). Both species have a worldwide distribution and can be found in natural and treated water, sewage, and dirt.^6^ Pathogenic RGM species primarily belong to the *M. fortuitum* group, the *M. chelonae/abscessus* group, or the M. smegmatis group.^6^ M. wolinskyi has been described as a distinct species within the *M. smegmatis group* in 1999.^7^ *M. mageritense* was defined as a new species in 1997. Due to its phenotype and biochemical properties (ability to utilize mannitol, inositol, and sorbitol) it was originally considered a member of the *M. fortuitum* third biovariant complex. Genetically it is, however, more closely aligned to the *M. smegmatis* group, and its 16S rRNA sequence differs by only 9 base pairs from that of *M. wolinskyi*.^3^^,^^6^

Infections with *M. wolinskyi* and *M. mageritense* have been observed in both overtly immunosuppressed and seemingly immunocompetent hosts.^5^ The hereditary predisposition to infections caused by weakly virulent mycobacteria in otherwise healthy individuals has been referred to as ‘Mendelian susceptibility to mycobacterial disease’. Several Mendelian susceptibility to mycobacterial disease-related genes, most of which are involved in the IFNγ-/interleukin-12 axis, have to date been identified. Identifiable genetic defects, however, account for only half of the known Mendelian susceptibility to mycobacterial disease cases.^1^^,^^2^ Autoantibodies to IFNγ have likewise been identified as a predisposing factor for nontuberculous mycobacterial infections. They are more prevalent in Asian populations,^8^ but have been ruled out in our present case. Given the exceedingly low prevalence of *M. wolinskyi/M. mageritense*-related diseases, we strongly suspect that some kind of either genetically defined or acquired immunodeficiency is a prerequisite for active clinical infection, even in patients lacking any previous indication of overt immunosuppression.

*M. wolinskyi* and *M. mageritense* are mainly associated with cutaneous infections, osteomyelitis, and bacteremia. Infections are usually preceded by surgery or penetrating trauma, and occasionally by cosmetic procedures.^3^, ^4^, ^5^ Our case of a spontaneous infection without a history of a preceding trauma represents an exception to this pattern. While it is the hitherto first description of a simultaneous infection with *M. wolinskyi* and *M. mageritense*, mixed infections of other nontuberculous Mycobacteria species are not uncommon and have been associated with environmental exposure.^9^ No environmental source, however, could be identified in the present case.

While RGM are commonly resistant to standard antituberculosis agents, no standardized treatment recommendations have to date been established. Though macrolides (eg clarithromycin) are considered appropriate to treat other nontuberculous mycobacterial infections, several RGM species including *M. mageritense* and *M. wolinskyi* are naturally resistant.^3^ Intrinsic macrolide resistance has been demonstrated to be conferred by the rRNA methylase erm(40) gene.^10^ Both *M. wolinskyi* and *M. mageritense* are commonly susceptible to amikacin, quinolones, and linezolid.^3^ Administration of at least 2 active antimicrobial agents is advocated.

## Conflicts of interest

None disclosed.
